# Diagnostic Challenges in the Myopathic Variant of Carnitine Palmitoyltransferase II Deficiency: A Case Report

**DOI:** 10.7759/cureus.64728

**Published:** 2024-07-17

**Authors:** Lana Alabbasi, Hadhami Ben Turkia, Maram Nass, Ibrahim Sahin

**Affiliations:** 1 Pediatrics, King Hamad University Hospital, Muharraq, BHR; 2 Medical Genetics, King Hamad University Hospital, Muharraq, BHR

**Keywords:** paediatrics disease, metabolic diseases, carnitine palmitoyltransferase ii deficiency, genetic diseases inborn, rhabdomyolysis

## Abstract

Carnitine palmitoyltransferase II deficiency is a rare metabolic disorder affecting the mitochondrial oxidation of fatty acids.

We present a case of the myopathic form in a 10-year-old Bahraini male following an initial presentation of exercise-induced rhabdomyolysis and transaminitis. There was no consanguinity or findings suggestive of an underlying inborn metabolic disorder. Tandem mass spectrometry on dried blood spots showed no abnormal acyl-carnitines profile. The condition improved with hyperhydration, high glucose intake, carnitine, and alkalinization. Genetic testing revealed a compound heterozygous pathogenic variant *c.338C>T* (*p.Ser113Leu*) and a variant of unknown significance *c.729_731del* (*p.Leu244del*). The patient was kept on a high carbohydrate and low-fat diet with medium chain triglycerides supplementation and advised to avoid long fasting periods and strenuous exercise. Within the four years of follow-up, he had three further attacks.

Exercise-induced myalgia or rhabdomyolysis should raise the suspicion of inherited metabolic disorders. Metabolic investigations should be taken during the acute illness, and an acylcarnitines profile should preferably be performed in the serum.

## Introduction

Mitochondrial beta-oxidation of fatty acids is one of the significant sources of energy, providing up to 80% of the total requirement during fasting or prolonged exercise. In such conditions, long-chain fatty acids (LCFAs), hexadecanoyl-L-carnitine (C16) to octadecenoyl-L-carnitine (C18) stored as triglycerides in fat tissue, are transported into cells and activated to acyl-CoA esters. The carnitine palmitoyltransferase II (CPT II) enzyme is an inner mitochondrial membrane protein that cleaves fatty acids from carnitine to be used in beta-oxidation resulting in acetyl-CoA production, which enters the Krebs cycle to produce energy or is converted to ketone bodies that can be used as alternative energetic fuel [1,2].

CPT II deficiency (CPT II-D) is an autosomal recessive disorder that can present with one of three phenotypes: lethal neonatal, infantile hepatocardiomuscular, or myopathic form. The former two types are severe multisystemic diseases characterized by hypoketotic hypoglycemia, cardiomyopathy, seizures, and early death. Around 20 families with the lethal neonatal form, 28 families with the severe infantile hepatocardiomuscular form, and 300 cases of the myopathic form have been reported. However, prevalence might be underestimated as many pregnancies with the lethal neonatal form end due to severe cerebral malformations, and symptoms of the myopathic form can be mild or may not occur at all leading to underdiagnosis [3]. The myopathic variant can manifest during childhood or early adulthood with exercise-induced myalgia and recurrent episodes of rhabdomyolysis (RM) of variable severity. The variant *p.Ser113Leu* represents about 70% of mutant alleles and is exclusively associated with the myopathic form [1].

We discuss here a challenging diagnosis of CPT II-D in a child after he presented with a severe first attack of exercise-induced RM. He carries the common *p.Ser113Leu* variant and a variant of unknown significance in the CPT II gene [4].

## Case presentation

A 10-year-old boy presented to the emergency department with a one-day history of lower limb pain that progressed to an inability to bear weight one hour after playing 90 minutes of football. He also complained of lower abdominal pain and noticed cola-colored urine. He denied other urinary symptoms, fever, or recent trauma, and this was his first episode.

He is a product of a non-consanguineous marriage and has five healthy siblings. There is no history of any genetic diseases running in the family. The birth history, growth, and development were normal. On examination, the patient was vitally stable and afebrile. His muscular power was five out of five in the upper and lower limbs, but could not bear weight because of the pain. Otherwise, there was no muscular tenderness, and deep tendon reflexes and sensations were intact. Urine analysis showed red urine with a white blood cell count of more than 50/hpf, red blood cell count of 11-20 /hpf, and negative nitrites. Blood tests showed high serum alanine aminotransferase (ALT) and aspartate aminotransferase (AST). Other liver parameters, complete blood count, C-reactive protein, renal function tests, electrolytes, and lactic acid were all normal (Table 1). The child was admitted initially under the impression of glomerulonephritis and liver derangement. The next day, creatine kinase (CK) came to be very high as well as creatinine kinase myocardial band (CK-MB) suggestive of RM (Table 1).

**Table 1:** Investigations.

	Test	Result	Reference value
Bloods	White blood count	11.42 × 10^9 ^/L	4 - 11× 10^9^/L
	Hemoglobin	13.8 g/dl	11 - 15.5 g/dl
	Platelets	275 × 10^9^/L	150 - 450 × 10^9^/L
	C-reactive protein	1 mg/l	0 - 10 mg/l
	Random glucose	6.9 mmol/l	3.6 - 8.9 mmol/l
	Sodium	138 mmol/l	137 - 148 mmol/l
	Potassium	3.6 mmol/l	3.5 - 5.1 mmol/l
	Urea	6.9 mmol/l	3 - 7 mmol/l
	Creatinine	60.5 umol/l	27 - 62 umol/l
	Total protein	72.3 g/l	64 - 82 g/l
	Albumin	43.4 g/l	30 - 50 g/l
	Globulin	28.9 g/l	23 - 35 g/l
	Bilirubin total	8.4 umol/l	1.71 - 20.5 umol/I
	Bilirubin direct	2.09 umol/l	0 - 5 umol/l
	Aspartate aminotransferase	972.1 u/l	10 - 40 u/l
	Alanine aminotransferase	331 u/l	16 - 63 u/l
	Alkaline phosphatase	241 u/l	10 - 14 years: 130 - 340 u/l
	Gamma-glutamyl transferase	20.7 u/l	0 - 35 u/l
	Hepatitis profile (A, B, and C)	Negative	Not applicable
	Activated partial thromboplastin ratio	0.94	0.9 - 1.4
	International normalized ratio	0.94	0.61 - 1.17
	Prothrombin time	11.4 seconds	10.7 - 13.9 seconds
	Lactic acid	1.8	< 2.15
	Calcium	2.1 mmol/l	2.12 - 2.62 mmol/l
	Magnesium	0.9 mmol/l	07 - 1 mmol/l
	Inorganic phosphorus	1.6 mmol/l	0.80 - 1.40 mmol/L
	Creatine kinase	155800 U/L	35 - 232 U/L
	Creatine kinase myocardial band (MB)	233.8 ng/ml	0 - 5 ng/ml
	Troponin-1	0.03 ng/ml	0.02 - 0.06 ng/ml
	Tandem mass spectrometry for amino acids, organic acids, and acylcarnitine	Normal	Not applicable
Urine	Urine culture and sensitivity	Negative	Not applicable
	Urine myoglobin	242 ng/ml	< 20 ng/ml

**Figure 1:**
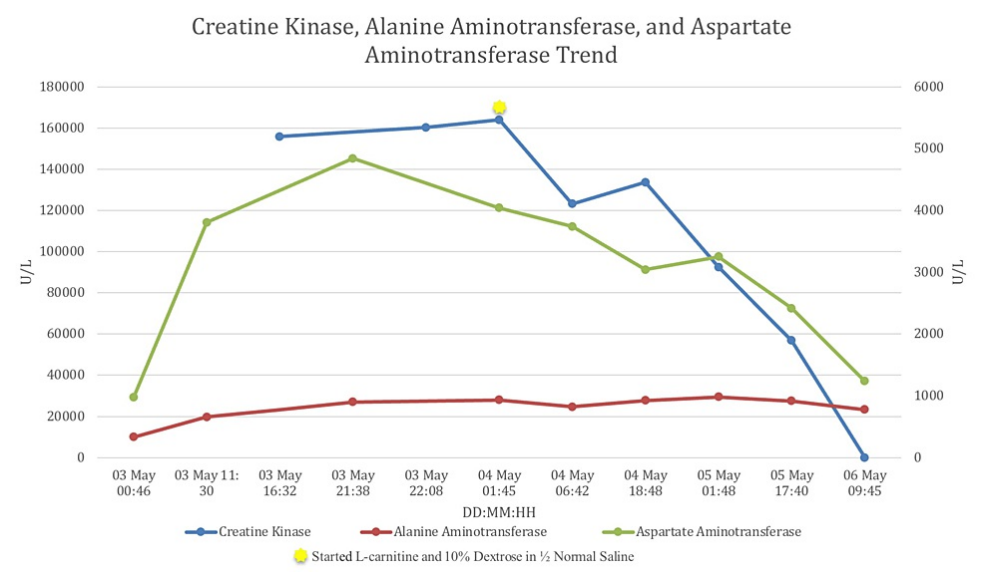
Creatine kinase, alanine aminotransferase, and aspartate aminotransferase trends.

In the following days, CK, AST, and ALT transaminases kept rising, reaching 164000 U/L, 4840 U/L, and 981 U/L, respectively (Figure 1), hence a fatty acid oxidation defect was suspected. The child was then started on one-and-a-half maintenance intravenous fluids with dextrose 10% normal saline providing a glucose infusion rate of 5.5 mg/kg/min along with an alkalinization and L-carnitine supplementation. RM workup included urine toxicology, high-performance liquid chromatography-tandem mass spectrometry (TMS) on dried blood spots, and urine amino acids. The ECG and echocardiogram were normal.

Subsequently, the child began to be active and pain-free. While the CK levels took three days to normalize, liver enzymes remained elevated for up to two weeks (Figure 1).

TMS was normal, urine amino acids showed elevated aspartic acid, threonine, serine, asparagine, glutamine, glycine, alanine, cystine, lysine, histidine, and arginine. Urine toxicology was negative. However, urine organic acids were not collected.

The AllNeuro (CentoGene, Rostock, Germany) panel has been performed and revealed compound heterozygosity for the pathogenic variant *c.338C>T* (*p.Ser113Leu*) and a variant of unknown significance (VUS) *c.729_731del* (*p.Leu244del*) in the CPT II gene. The VUS was described as an in-frame deletion of three bps in exon four, which causes the loss of residue Leu at position 244. Each mutation and its pathogenicity were validated by bioinformatics analysis, Sanger sequencing-based co-segregation testing, and computational assessment. Computational methods have demonstrated their association with disease.

A high carbohydrate, low-fat diet, daily medium chain triglycerides (MCT) oil supplementation, and carnitine were prescribed. The child was advised to avoid fasting for more than 12 hours and to take a snack and an extra MCT dose 30 minutes before exercise.

During his follow-up, the patient continued to experience intermittently some degree of leg pain after walking even though his CK levels were normal. He was found to have low vitamin D and was given vitamin D supplements.

In four years, the patient experienced three more episodes of RM due to non-compliance to dietary treatment, which required admission. Since then, he has avoided any intensive physical activity.

## Discussion

RM is a clinical condition caused by skeletal muscle breakdown due to multiple etiologies. These causes include severe trauma, vigorous exercise, burns, electrical injury, seizure, electrolyte imbalances, drugs, infectious diseases, genetic neuromuscular disorders, and metabolic myopathies (MMs) [5].

With the release of muscular intracytoplasmic proteins, a risk of life-threatening complications may develop such as hyperkalemia, hypocalcemia, hypovolemia, acute kidney injury, compartment syndrome, and rarely disseminated intravascular coagulation. RM should be suspected in patients presenting with a triad of myalgia, weakness, and reddish-to-brown urine. Elevated serum CK levels over 1000 U/L indicate RM [6]. In our patient, CK level reached 700 x upper limit of normal, which could have exposed the child to life-threatening complications due to a delay in the management on admission. Despite displaying the full triad of RM, our patient was initially misdiagnosed as acute hepatitis because of markedly elevated liver enzymes; however, an AST-to-ALT ratio of > 2:1 is suggestive of muscle injury rather than hepatitis [7].

Inherited metabolic disorder (IMD) as a cause of RM is often missed; in 475 patients admitted for RM, 10% were related to underlying genetic or MM [8]. MM can be caused mainly by glycogen storage disorders, fatty acid oxidation defects (FAODs), or mitochondrial disorders. Other MMs are listed in Table 2 [5,7,9].

**Table 2:** Inborn errors of metabolism causing rhabdomyolysis. Reproduced from Yazıcı H, Kalkan Uçar S: A metabolism perspective on pediatric rhabdomyolysis. Trends in Pediatrics. 2021, 2:147-53 [5]. Permission was obtained from Dr. Havva Yazıcı.

Inborn errors of metabolism causing rhabdomyolysis	
Glycogen storage disorders	Glycogen storage disease type V
Disorders of glycolysis	Aldolase A deficiency
	Lactate dehydrogenase deficiency
	Muscle phosphofructokinase deficiency
	Phosphoglycerate kinase deficiency
Disorders of mitochondrial fatty acids oxidation	Carnitine palmitoyltransferase 2 deficiency (CPT2)
	Long-chain 3-hydroxyacyl-CoA dehydrogenase deficiency (LCHAD)
	Very long-chain acyl-CoA dehydrogenase deficiency (VLCAD)
	Trifunctional protein deficiency (TF)
Mitochondrial disorders oxidative phosphorylation system deficiencies	
Others	Lipin-1 deficiency (LPIN1)
	Transport and Golgi organization 2 (TANGO2)
	Ryanodine receptor 1 (RYR1)

Physicians should have a high degree of suspicion of an underlying IMD if there is a personal or family history of recurrent RM or exercise intolerance (cramps, myalgia) [10]. It is important to note that the presence of an identifiable trigger does not necessarily exclude an underlying genetic cause [11]. As in our case, the patient denied any history of exercise intolerance before the first attack.

In long-chain fatty acid oxidation disorders (LCFAODs), attacks or myoglobinuria occur typically after mild to moderate prolonged exercise or when patients are additionally stressed by fasting, cold exposure, or infection. RM may present as either isolated or in association with life-threatening manifestations such as hypoglycemia, cardiomyopathy, arrhythmia, liver impairment, and/or encephalopathy [12].

The myopathic form of CPT II-D is the most common lipid metabolism disorder affecting skeletal muscle [10]. Even though the myopathic form is also called the adult form, 60% of patients manifested their symptoms below the age of 12 years [1].

In contrast to patients with other MMs, no residual weakness or persistent hyperCKemia is seen between the attacks in CPT II-D whereas only 10% of affected individuals have been observed with a consistent elevation of serum CK level [5,13].

The diagnosis of CPT II-D can be challenging. The acylcarnitines analysis performed on dried blood spots (DBS) for our patient in the first two attacks did not show the typical profile of elevated C16 and C18:2 species, and the increased C16+ C18:1/C2 ratio [14]. This test should be performed in plasma or serum as the diagnosis can be missed when it is performed in DBS [15]. Furthermore, normal plasma acylcarnitines do not rule out the diagnosis even during the acute attack. Urine organic acids might show non-specific dicarboxylic aciduria. The generalized aminoaciduria observed in our patient was related to muscle protein release.

FAOD study can be performed on fibroblasts or muscle tissue; however, molecular genetic testing has become the gold standard for establishing a definitive diagnosis [1].

Due to the broad clinical and genetic variability of FAOD, diagnosing them quickly and precisely is challenging. To date, 179 likely or pathogenic mutations have been reported in association with the myopathic form [16].

In the present case, compound heterozygous mutations have been found. The *p.Ser113Leu* variation accounts for approximately 70% of mutant alleles and is linked explicitly to the myopathic type [1,17]. The protein length changing *c.729_731del* (*p.Leu244del*) mutation belongs to evolutionary highly conserved regions. Silico tools showed that the mutation was disease-causing. This variant has been reported in one patient with the lethal neonatal form [4].

It is advised to evaluate even asymptomatic relatives to reduce the morbidity and mortality rate. This is particularly crucial before any general anesthesia, which might expose to severe RM [3].

Acute management consists of early fluid resuscitation with dextrose 10% at a rate of one and a half to double maintenance to enhance the anabolism and ensure sufficient urine output [12]. The use of carnitine is controversial during acute attacks of LCFAOD [18].

The dietary management of LCFAOD consists of a high carbohydrate intake covering 60% of the total energy requirement. Fat intake should be limited to 30-35%, with long-chain fat restricted to only 15% of the total energy. MCT supplementation covers the remainder of the energy requirement. Patients should avoid identifiable triggers, such as extended fasting and prolonged exercise. Moreover, a low-fat snack with MCT oil should be provided 30 to 45 minutes before strenuous exercise. Patients should also be monitored for secondary carnitine deficiency [12].

## Conclusions

This case report highlights the importance of metabolic and genetic testing in patients presenting with RM. Although the clinical presentation of myopathic CPT II-D was typical, TMS on DBS was not diagnostic. The physician should inquire about personal or family history of exercise-induced myalgia or intolerance and collect critical samples before starting hyperhydration with high glucose concentration fluid. Genetic testing remains the gold standard diagnostic test. Our data offer novel perspectives for understanding the relationship between genotypes and phenotypes in CPT II-D. Further functional studies should be undertaken to investigate the effects of mutations on the disease.
